# Nanofiber template-induced preparation of ZnO nanocrystal and its application in photocatalysis

**DOI:** 10.1038/s41598-021-00303-9

**Published:** 2021-10-27

**Authors:** Mingyi Chen, Peng Liu, Ji-Huan He, Hsing-Lin Wang, Haonan Zhang, Xin Wang, Rouxi Chen

**Affiliations:** 1 Department of Materials Science and Engineering, Southern University of Science and Technology, Shenzhen, 518055 China; 2 National Engineering Laboratory for Modern Silk, College of Textile and Clothing Engineering, Soochow University, Suzhou, 215123 China; 3 Songshan Lake Materials Laboratory, Dongguan, 523808 China

**Keywords:** Nanowires, Organic-inorganic nanostructures, Photocatalysis

## Abstract

Traditional preparation of ZnO nanocrystal requires heating zinc acetate to a temperature over 350 °C, whereas in this work, zinc acetate was first electrospun with PVDF to form a nanofiber, followed by thermal treatment at only 140 °C to give nanocrystalline ZnO. The much lower temperature required in thermal treatment is attributed to the high reactivity of zinc acetate at nano dimension. The as-prepared ZnO-doped PVDF nanofiber mat shows excellent effect in the photocatalytic degradation of Rhodamine B, comparable to ZnO particle thermally treated at 600 °C. Highly-oriented ZnO nanorods were obtained by further hydrothermal synthesis of the electrospun nanofiber mat, giving nanostructured ZnO of different morphologies well-aligned on the surface of organic nanofiber. Notably, the hydrothermal synthesis of the successful preparation of these nanostructured ZnO requires a processing temperature below 100 °C at atmospheric pressure, showing great potential to be scaled up for vast manufacturing.

## Introduction

Nanostructured materials possess unique properties because their constituent units are at a dimension comparable to the size of molecules. Nanofibers are flexible materials that are bendable and knotable, making them perfect candidates for wearable devices. Compared to pristine ZnO particle, the organic nanofiber composites loaded with nano ZnO are of great importance, with excellent optical, electrical, mechanical and chemical properties, enabling many potential applications in optoelectronic devices^1–3^, sensors^4^, photocatalysts^5–8^, etc.

There are a few ways for the preparation of ZnO nanomaterials, such as gas-phase synthesis^9–12^, solid-phase^13^ based synthesis and liquid-phase synthesis. Although these syntheses have succeeded in the preparation of nano ZnO with various morphologies, costly equipment with strict synthesis conditions, such as high temperature and high pressure, are often required. Moreover, obtaining organic nanofiber materials loaded with highly oriented ZnO is also challenging with these synthetic methods. In order to optimize the synthesis, in this work, electrospinning was employed to prepare nanofiber mat precursors, followed by low-temperature thermal treatment to give organic fibers with ZnO nanoparticles^14–18^, and finally hydrothermal preparation, leading to highly ordered ZnO nanorods nanofiber mat. This cost-efficient approach requires very little on the equipment thus can be readily scaled up for mass production.

## Experimental

### Materials

The materials used in this study are listed in Table S1. All materials were all used as received unless otherwise indicated. A polyvinylidene fluoride (PVDF) polymer with a molecular weight of 400,000 g/mol was used as the main constituent for electrospun nanofiber template. The relatively high molecular weight of the polymer ensures structural and thermal stability when subjected to heat treatment in the preparation of ZnO crystals.

### Preparation and treatment of nanofiber

#### Preparation of PVDF/Zn(Ac)_2_ nanofiber mat

A certain amount of PVDF particles and a mixed solvent of N–N dimethylformamide (DMF) and acetone were added in a beaker. The solution was heated and kept at 55 °C. Anhydrous zinc acetate (Zn(Ac)_2_) powder was added to the beaker when the PVDF particles were completely dissolved. The solution was stirred until a uniform dispersion solution was formed. PVDF/Zn(Ac)_2_ nanofiber mats were then prepared by electrospinning at a voltage of 15 kv and a receiving plate-to-spinning end distance of 12 cm. The recipe of the spinning solution is shown in Table S2.

#### Low temperature treatment for the preparation of ZnO-loaded PVDF nanofiber mat

*Experiment 1* The PVDF/Zn(Ac)_2_ nanofiber mat was thermally treated from 60 to 200 °C at an interval of 20 °C for 24 h.

*Experiment 2* The PVDF/Zn(Ac)_2_ fiber mat was thermally treated at 140 °C for 1 h, 12 h, and 36 h.

#### Preparation of PVDF/ZnO nanorods mat

First, 60 mL of deionized water and desired amount of zinc chloride (ZnCl_2_) powder was placed in a beaker and stirred for 10 min, then hexamethylenetetramine (HMTA) powder was added and stirred till complete dissolution. To this solution, a certain volume of ammonia solution was added, then ionized water was filled to make a total solution volume of 100 mL. Second, the PVDF/Zn(Ac)_2_ nanofiber mats thermally treated at 140 °C were subjected to hydrothermal reaction to allow the growth of ZnO nanorods. Finally, the ZnO-loaded PVDF nanofiber mats were rinsed several times with deionized water, and dried in a muffle furnace.

#### Factors affecting the growth of ZnO nanorods

The influence of the M(ZnCl_2_:HMTA) molar ratio on the structure of ZnO nanorods was studied. Four M(ZnCl_2_:HMTA) ratios were employed: 1:3, 1:2, 1:1 and 2:1. The 100 mL solution with 5 mL of ammonia was subject to hydrothermal reaction for 3 h.

The influence of hydrothermal temperature on the nanostructure of ZnO was investigated with a ZnCl_2_:HMTA molar ratio of 1:1 at a total concentration of 0.1 M. The solution, with 5 mL of ammonia and a total volume of 100 mL, was subject to hydrothermal reaction at night different temperatures for 3 h.

Influence of volume of ammonia solution on the nanostructure of ZnO was studied. The solution with a growing solution concentration of 0.1 M and a ZnCl_2_:HMTA molar ratio of 1:1 was filled with different volume of ammonia for a total solution volume of 100 mL. The solution was subject to hydrothermal reaction at 90 °C for 3 h.

The nanostructure of ZnO prepared from different growing time was compared. The solution with a growing solution concentration of 0.1 M and a ZnCl_2_:HMTA molar ratio of 1:1 was filled with 5 mL of ammonia to a total volume of 100 mL, followed by hydrothermal reaction at 90 °C for different time.

The morphologies of ZnO prepared on nanofiber mat are pre-treated for 18 h at different temperature. The solution with a growing solution concentration of 0.1 M and a ZnCl_2_:HMTA molar ratio of 1:1 was filled with 5 mL of ammonia to make a total volume of 100 mL, followed by hydrothermal reaction at 90 °C for 3 h.

#### Photocatalytic investigation

##### Experiment 1

1 g of the untreated PVDF/Zn(Ac)_2_ fiber mat, the PVDF/Zn(Ac)_2_ fiber mat thermally treated at 140 °C and the ZnO powder calcined at 600 °C were weighed and added to 3 parts of rhodamine B solution with a concentration of l0 mg/L and a total volume of 30 mL. Then the above rhodamine B solution was placed in dark for 1 h for adsorption desorption equilibrium treatment, and the photocatalytic degradation experiment was carried out under the irradiation of ultraviolet (20 W, 254 nm), and the blank control was carried out (Note: in the process of ultraviolet irradiation, the fiber mat is laid flat in the solution, and the powder is statically dispersed in the solution). The photocatalytic effect was recorded by taking the supernatant every 30 min and measuring the absorbance of the sample with an UV–Vis spectrophotometer at the maximum absorption wavelength of rhodamine B (554 nm).

##### Experiment 2

The thermally treated PVDF/Zn(Ac)_2_ fiber mat (3 g) with ZnO nanorods of different weights (1 g, 2 g and 3 g) and 1 g of pure ZnO powder synthesized by hydrothermal method were added to a 10 mg/L rhodamine B solution to make a total solution volume of 30 mL. The rest of the experiment is the same as experiment 1.

##### Experiment 3

2 g of ZnO nanorod-loaded PVDF/Zn(Ac)_2_ nanofiber mats hydrothermally synthesized with different reaction time (1 h, 3 h and 5 h) were added to a 10 mg/L rhodamine B solution to make a total solution volume of 30 mL. The rest of the experiment is the same as experiment 1.

## Results and discussion

### Preparation of PVDF/Zn(Ac)_2_ nanofiber mat

In the preparation of PVDF/Zn(Ac)_2_ fiber mat by electrospinning, the concentration of the spinning solution and the content of zinc acetate in the spinning solution have a significant influence on the morphology and properties of the final product.

#### Effect of polymer solution concentration on the structure of nanofibers

The nanofiber mat prepared with 5% concentration of PVDF has a large number of irregular bead structure fibers with basically no fibrous form of filaments, as seen from Figure S1. As the concentration increases to 10%, even and complete fibers with an average fiber diameter of 133 ± 33 nm can be obtained, despite a small amount of beaded fibers presented. The fibers with uniform thickness and smooth surface were obtained with a 15% PVDF concentration. The fiber has basically no bead structure with an average diameter of 366 ± 67 nm. At a PVDF concentration of 19%, the surface of fibers becomes rough and the thickness of a single fiber is uneven along the axial direction. The average diameter of the fiber spun from the 19% PVDF solution is 832 ± 144 nm, much larger than those from a 15% PVDF solution.

The variation of the fiber diameters and morphology is mainly because of the difference in viscosity and surface tension of the spinning solution. When the viscosity is small, the electrostatic force on the liquid column is much greater than its surface tension. Therefore, the liquid column cannot be quickly stretched and thinned in a very short time, but is rather directly pulled off to form an irregular spindle bead structure. On contrary, when the viscosity and surface tension of the spinning solution are too large, the electrostatic force posed on the solution is not strong enough to overcome the surface tension of the liquid column, resulting in the disorder of stretching and refining of the liquid column thus leads to the uneven thickness and rough surface of a single fiber.

#### Effect of zinc acetate content in solution on the structure of nanofibers

When a small amount of zinc acetate is added, the morphology and structure of the fiber have only little change, as shown in Figure S2a. Further increasing the content of zinc acetate did not make a difference on the morphology and structure of the fiber, as shown in Figure S2b, At this relatively larger zinc acetate content, a few and fine short fibers are produced and the fiber diameter becomes smaller. When the content of zinc acetate is too large, not only the surface roughness of the fiber increases, but a large number of very fine short fibers are produced, and the diameter of the fiber becomes significantly uneven, as shown in Figure S2c; When the zinc acetate content reaches saturation in the solution, the uniformity of the fiber is improved due to incomplete dissolution and continuous deposition during spinning, as shown in Figure S2d. In addition, the average diameters of PVDF/Zn(Ac)_2_ nanofibers prepared with precursor solutions of N31, N32, N33 and N34 in Table S2 are 446 ± 65 nm, 366 ± 67 nm, 509 ± 138 nm and 458 ± 83 nm, respectively. It can be seen that the nanofibers prepared by a PVDF/Zn(Ac)_2_ molar ratio of 10:3 have the best fibrous morphology.

This much improved morphology is mainly because of the ionic nature of zinc acetate which exists in the form of ions in solution. When added a small amount of zinc acetate, the concentration and viscosity of the solution only increase slightly, while the electric conductivity increases significantly. During spinning, the electrostatic force enhancement rate of the liquid column is greater than its surface tension, and the fiber diameter decreases gradually. When added a large amount of zinc acetate, the concentration and viscosity of the solution increase significantly, resulting in a high electric conductivity. In such case, the solution can easily become a sol, making the spinning electrically unstable.

### Preparation of ZnO nano seed loaded on PVDF nanofiber mat

Solutions of inorganic salt are usually not suitable for electrospinning of nanofiber because they do not have appropriate concentration, viscosity and surface tension that are required by the technique for continuous long-range fibers. Instead, a PVDF solution pre-dissolved with zinc acetate (Zn(Ac)_2_) was successfully used for electrospinning, giving a PVDF/Zn(Ac)_2_ nanofiber mat. Simple thermal treatment of the as-prepared nanofiber mat leads to ZnO-loaded PVDF nanofiber. The nanofiber mat prepared under N32 conditions were selected for heat treatment.

#### Effect of heat treatment temperature on the structure of PVDF/Zn(Ac)_2_ nanofibers

As shown in Figures S3 and S4, the surface of the untreated PVDF/Zn(Ac)_2_ nanofibers is relatively smooth. The fiber is homogeneous, with uniformly distributed substances inside the fiber, which shows that zinc acetate is evenly dispersed on the PVDF fiber. After heat treatment at 80 °C, a few particles start to appear on the fiber surface, and the fiber surface begins to become rough, while the number of dispersions decreases and changes to aggregation state, as seen in the corresponding TEM diagram, indicating that the dispersed zinc acetate in the fiber may be subject to a certain change at 80 °C. After thermal treatment at 100–160 °C, a large number of particles on the fiber surface is clearly seen, where the particles gradually increase in number. The particle size is gradually increasing, along with increased surface roughness of fiber and more obvious uneven size distribution. In addition, large particles appear at 140 °C and above. At 140 °C, the fiber can preserve the morphology and structure, while at 160 °C, A few micro cracks began to appear on the fiber surface, and the morphology and structure of the fiber began to collapse. Above 180 °C, a large number of cracks present on the rough fiber surface, and the particles on the fiber surface are reduced. The TEM images show that there are many grooves on the fiber with a large number of particles inside the fiber. After heat treatment at 200 °C, the fiber mat is completely melted with no fibrous structure left^19,20^. A large number of discrete particles are distributed in the melted mat, as seen from the TEM image, which shows that heat treatment at 180 °C and above will destroy the PVDF fiber mat template. Lastly, 140 °C was found to be the optimal heat treatment temperature for PVDF/Zn(Ac)_2_ nanofibers, which can give a large number of ZnO nano seeds and preserve the complete morphology and structure of the fibers.

#### Effect of heat treatment time on the structure of PVDF/Zn(Ac)_2_ nanofibers

After heat treatment for 1 h, a small amount of fine particles were produced on the rough fiber surface, suggesting the change of zinc acetate in this process, as shown in Figure S5. After heat treatment for 12 h, a large number of fine particles are produced on the fiber surface. Compared to the fiber thermally treated for 1 h, the one treated for 12 h have clearly larger particle size and rougher surface, which indicates that zinc acetate will further transform into larger particles in a longer heat accumulation process. After heat treatment for 36 h, no further increase in the number and size of particles on the fiber was observed, indicating possible saturation in conversion of zinc acetate in the fiber after ultra-long heat treatment.

Although the duration of heat treatment has a great influence on the size and quantity of ZnO nano seed in PVDF/Zn(Ac)_2_ nanofiber mat, the effect of heat treatment temperature on the formation of ZnO nano seed and the morphology and structure of fibers in PVDF/Zn(Ac)_2_ nanofiber mat is more critical.

### Preparation of ZnO nanorods loaded on PVDF nanofiber mat

The ZnO nanorods were prepared by hydrothermal synthesis of the ZnO nanoseed-loaded PVDF nanofiber mat, thermally treated at 140 °C, in a growth-promoting solution consisting ZnCl_2_, hexamethylenetetramine (HMTA) and ammonia.

#### Effect of growth solution concentration on ZnO nanorods

When m (ZnCl_2_:HMTA) in the growth solution is 1:3 and 1:2, the prepared ZnO nanorods appear to be irregular spire hexagons that are uneven in length, as shown in the Figure S6. Moreover, the orientation of ZnO nanorods on the fiber mat is disordered, with nearly all growth appears only on the surface of fibers. When m (ZnCl_2_:HMTA) in the growth solution is 1:1, the prepared ZnO nanorods have regular hexagonal shapes without any spires. The orientation of ZnO nanorods on the fiber mat is relatively neat. Besides, ZnO nanorods are also growing on the inner zone of the fiber mat, where the nanorods are finer than those from the outer layer. When m (ZnCl_2_: HMTA) in the growth solution is 2:1, flake zinc oxide is obtained instead of rod zinc oxide. The flake zinc oxide does not grow evenly on the fiber, and the distribution is also very disordered. Therefore, a m (ZnCl_2_: HMTA) of 1:1 was finally adopted for the growth solution, which gives ZnO nanorods in uniform vertical orientation growing on the fiber surface.

#### Effect of hydrothermal reaction temperature on ZnO nanostructures

The SEM images of the ZnO nanostructure obtained from different hydrothermal temperature are shown in Figure S7. When the hydrothermal reaction temperature is 50 °C, the synthesized single ZnO nanorods appear to be like corn rods with very rough surface. These nanorods present on the fiber surface with very irregular orientation, forming a flower-like structure. The length of the synthesized ZnO nanorods is very short with an average diameter of about 162 ± 19 nm. When the hydrothermal reaction temperature rises to 60 °C, the structure of ZnO nanorods becomes scattered hexagons with smooth surface and more uniform orientation. The average diameter of ZnO also increases significantly to 288 ± 31 nm. With further increase of hydrothermal reaction temperature to 70–90 °C, the synthesized ZnO nanorods evolve from spire hexagonal prism to flat top hexagonal prism. The average diameter of ZnO nanorods is about 300 nm and the vertical orientation of ZnO nanorods on the fiber also becomes more regular. When the hydrothermal reaction temperature reaches 100 °C, the ZnO nanorods are very regular flat topped hexagonal prisms with significantly decreased diameter of about 159 ± 21 nm. The difference of morphology and structure reaches the minimum, and the vertical orientation is very regular. At 110 °C, 120 °C and 130 °C, the synthesized ZnO nanorods can grow with a fairly good vertical orientation on the fiber surface, but the average diameter of the nanorods gradually increases with the increase of temperature, and the uneven diameter distribution also increases. The average diameters of the ZnO nanorods synthesized at the three temperatures are 227 ± 38 nm, 280 ± 32 nm and 321 ± 46 nm, respectively. When the hydrothermal reaction temperature reaches 140 °C, the top of ZnO nanorods becomes a smooth cap whose diameter increases significantly to about 437 ± 59 nm.

#### Effect of ammonia volume on ZnO nanostructures

The influence of volume of ammonia solution on the nanostructure of ZnO was studied, as shown in Figure S8. The hydrothermal synthesized zinc oxide in the growth solution without ammonia is in sheet shape and does not grow on the fiber surface. The zinc oxide synthesized by hydrothermal synthesis with 1 mL of ammonia solution is a mixture of flake and rod, and the growth of zinc oxide on the fiber mat is very chaotic. The growth solution added with 2 mL of ammonia was hydrothermally synthesized to give spindle ZnO nanorods. Here, crystalline ZnO preferentially grows on the surface of the fiber in a disordered vertical orientation, forming short yet fine nanorods with an average diameter of 134 ± 16 nm. ZnO nanorods with uniform diameter were hydrothermally synthesized by adding 3 mL and 4 mL of ammonia solution. The morphological structure gradually became clear shuttle hexagonal prism, and the vertical orientation on the fiber surface became regular. Moreover, the longitudinal length and diameter of the synthesized ZnO nanorods increased significantly, and their average diameters were measured to be 289 ± 32 nm and 405 ± 31 nm, respectively.

When the amount of ammonia was 5 mL, the synthesized ZnO nanorods evolved from spindle hexagonal prism structure to regular hexagonal prism structure, and the vertical growth orientation on the fiber was further enhanced. The diameter of ZnO nanorods decreased significantly, and its average diameter was 272 ± 29 nm. When ammonia is further increased to 7 mL, the synthesized zinc oxide shows a sheet structure that forms disorderly on the surface of the fiber, where the growth of adjacent nano sheets will affect each other.

#### Effect of growth time on ZnO nanostructures

The nanostructure of ZnO prepared from different growing time was compared, as shown in Figure S9. It was seen that when processed for 1 h, ZnO nanorods show spindle hexagonal prism structure that are vertically oriented on the fiber. The surface of ZnO nanorods is rough that filled with multi-particles. The nanorods are short that closely arranged only on the surface of fiber. After 3 h of growth, ZnO nanorods become flat topped, similar to regular hexagonal prism structure. This structure remained stable even with longer time of processing, but the difference in the hexagonal prism structure and in the longitudinal size both became larger. After the arrangement of ZnO nanorods on the same support fiber gradually diverges, ZnO nanorods on different fibers become dense, due to their significant overlapping.

#### Effect of pre-treatment temperature on the ZnO nanostructures

The morphologies of ZnO prepared on nanofiber mat pre-treated for 18 h at different temperature are shown in Figure S10. The ZnO crystals grown on the untreated electrospun PVDF/Zn(Ac)_2_ fiber mat is a mixed structure of nanorods and nanosheets. ZnO nanosheets are disorderly attached to the fiber surface, and ZnO nanorods are randomly disorderly attached to the fiber or nanosheets, which indicates that the ZnO nanorods synthesized by hydrothermal method cannot grow in an orderly vertical orientation on the fiber surface without zinc oxide seeds. However, with large amount of Zn(Ac)_2_, ZnO may be generated by hydrolyzation in hydrothermal reaction, which provides the possibility for the growth of ZnO nanosheets on the fiber. For ZnO nanorods grown on PVDF/Zn(Ac)_2_ fiber mat pre-treated at 60 °C, the crystals are found to be attached to the fiber surface in a disordered manner instead of extending vertically from the fiber surface, showing that the heat treatment at 60 °C cannot convert zinc acetate into ZnO particles. In contrast, ZnO grown on PVDF/Zn(Ac)_2_ fiber mat pre-treated at 80 °C and 140 °C show well-aligned rod morphology vertical to the surface of the fiber, indicating the starting temperature of the transformation of about 80 °C. This starting temperature is further supported by the fact that only a small number of ZnO nanorods was found to form on the surface of the fiber mat treated at 80 °C, whereas a large number of ZnO nanorods were found in the inner layer of the fiber mat treated at 140 °C. ZnO nanorods formed on the PVDF/Zn(Ac)_2_ fiber mat treated at 200 °C are in a disordered orientation that are not completely attached to the support fiber. This is mainly because at 200 °C, the fiber has completely melted and become a plastic paper mat where the ZnO particles are unable to evenly distribute in the plastic mat as in a fiber mat. Under the same growth conditions, the structure of zinc oxide grown on aluminum foil paper is flake. In comparison, zinc oxide was found to form nano sheets on the surface of aluminum foil in a regular orientation, as seen in Figure S10f.

The concentration of the growth-promoting solution, proportion of ammonia, time and temperature of thermal synthesis, temperature of thermal treatment all have an influence on the growth of ZnO nanorods. The results show that the best orientation of the ZnO nanorods were obtained at a growth-promoting solution concentration of 0.1 M with ZnCl_2_:HMTA molar ratio of 1:1 and 5 mL of ammonia. ZnO nanorods with the smallest diameter are obtained at a hydrothermal temperature of 100 °C, while well-dispersed and uniformly structured ZnO nanorods are achieved when the time of hydrothermal synthesis is 3 h. The nanofiber mat of PVDF/Zn(Ac)_2_ without pre-thermal treatment mainly consists interstacked ZnO crystals of mat shape. In comparison, the one thermally treated at 140 °C has well-defined nanorods that are aligned along the c axis.

### Growth mechanism

#### Growth mechanism of ZnO nano seed

The formation of ZnO nano seed is through a series of thermal degradation and chemical reaction. In the PVDF solution with mixed solvent of acetone and DMF, zinc acetate can combine with water in the solution or complex with polymer to produce partial double hydrolysis reaction, giving Zn(OH)_2_, that goes into the polymer fiber through electrospinning. After heat treatment at low temperature (140 °C), Zn(OH) _2_ in the fiber is heated and decomposed into ZnO, as shown in Figure S11. At the same time, zinc acetate on the fiber surface may also absorb water in the air or directly combine with water in the air. Under heating, it continues hydrothermal reaction and decomposes to form ZnO nanocrystals, as can be seen in the DSC thermograms of the PVDF/Zn(Ac)_2_ in Figure S12. The chemical equation of ZnO nanocrystalline mechanism formed by heating of zinc acetate can be expressed as follows:

$$\begin{gathered} {\text{Zn}}\left( {{\text{CH}}_{3} {\text{COO}}} \right)_{2} \left( l \right) + {\text{H}}_{2} {\text{O}}\left( l \right)\mathop{\longrightarrow}\limits^{hydolysis}{\text{Zn}}\left( {{\text{OH}}} \right)_{2} \left( s \right) + {\text{CH}}_{3} {\text{COOH}}\left( l \right) \hfill \\ \mathop{\longrightarrow}\limits^{heat}{\text{ZnO}}\left( s \right) + {\text{H}}_{2} {\text{O}}\left( g \right) + {\text{CH}}_{3} {\text{COOH}}_{2} \left( g \right) \hfill \\ \end{gathered}$$

#### Growth mechanism of ZnO nanorods

ZnO nanorods were grown on fibers with ZnO nano seed by hydrothermal method. The mixed solution of zinc nitrate (or zinc acetate, zinc chloride), hexamethyltetramine (HMTA) and ammonia as the growth solution had a complex chemical reaction. HMTA hydrolyzed in the solution to release OH^−^ ions, and ammonia ionized in the solution to release OH^−^ ions. The OH^−^ ions can combine with Zn^2+^ ions through complexation, forming [Zn(NH_3_)_4_]^2+^ or [Zn(OH) _4_]^2−^ ions, which further nucleate and give ZnO nanorods on the fiber surface after hydrothermal dehydration at elevated temperature, as shown in Figure S13 the XRD diffractograms of nanofiber mat loaded with Zn(Ac)_2_ under different thermal treatment, where the crystalline structure is clearly seen. The growth mechanism of ZnO nanorods can be expressed as follows^21–23^:

$$(\text{CH}_{2} )_{6} \text{N}_{4} + 6\text{H}_{2} \text{O} \leftrightarrow 4\text{NH}_{3} + 6\text{HCHO}$$

$$\text{NH}_{3} + \text{H}_{2} \text{O} \leftrightarrow \text{NH}_{3} \cdot \text{H}_{2} \text{O} \leftrightarrow \text{NH}_{4}^{ + } + \text{OH}^{ - }$$

$$\text{Zn}^{2 + } + \text{NH}_{3} \leftrightarrow [\text{Zn}(\text{NH}_{3} )_{4} ]^{2 + }$$

$$\text{Zn}^{2 + } + 4\text{OH}^{ - } \leftrightarrow [\text{Zn}(\text{OH})_{2} ]^{2 - }$$

$$\text{Zn}^{2 + } + 2\text{OH}^{ - } \leftrightarrow \text{Zn}(\text{OH})_{2} \leftrightarrow \text{ZnO} + \text{H}_{2} \text{O}$$

### Study on photocatalysis

As shown in Figure S14, pure rhodamine B solution degrades by 13% in 4 h under UV irradiation without any catalysts. The rhodamine B solution with the untreated PVDF/Zn(Ac)_2_ nanofiber mat, the PVDF/Zn(Ac)_2_ nanofiber mat after heat treatment at 140 °C and the ZnO powder calcined at 600 °C degraded about 21%, 39% and 47% respectively with 4 h of UV irradiation at a proportionally increased degradation rate. It can be seen that the UV catalytic degradation rate of untreated PVDF/Zn(Ac)_2_ nanofiber mat is only slightly higher than that of neat rhodamine B. This may be caused by the adsorption of rhodamine B by the fiber mat, indicating that the untreated PVDF/Zn(Ac)_2_ nanofiber mat has no obvious degradation effect on rhodamine B. The PVDF/Zn(Ac)_2_ nanofiber mat after heat treatment and the ZnO powder obtained by calcination obviously accelerate the UV catalytic degradation of rhodamine B, with a little difference in the degradation effect, which indicates that ZnO nano seeds may be distributed on the fiber mat as photocatalysts after heat treatment. Due to the uneven distribution in rhodamine B solution and inevitable precipitation, ZnO powder cannot provide a more efficient catalytic ability.

The photocatalytic degradation of rhodamine B by ZnO nanorods was studied, as shown in Figures S15 and S16. It is found that the catalytic degradation efficiency of ZnO nanorods grown on fibers is higher than that of ZnO nanorod powder without the polymer fiber support. The higher the ZnO content, the higher the catalytic efficiency. This may be because ZnO powder is easy to deposit at the bottom of the test tube during static adsorption degradation in the solution. The highly oriented ZnO nanorods grown on the fiber mat effectively increase the receiving area of photocatalysis, which effectively increase the UV absorbance that facilitate the photocatalytic degradation. For the fiber mat of ZnO nanorods prepared with the same content and different hydrothermal growth duration, the results show that the ZnO nanorods with a growth duration of 3 h have the highest catalytic degradation efficiency, while either shorter or longer hydrothermal treatment does not give an efficient catalytic degradation of rhodamine B. This may be due to the relatively short ZnO nanorods obtained from 1 h of hydrothermal growth, which are closely arranged on the fiber, allowing growth of subsequent nanorods only on the surface of fiber. The ZnO nanorods obtained by 3 h of hydrothermal growth are long and regular in morphology and structure, with orderly orientation and uniform dispersion on the fiber. In contrast, the ZnO nanorods obtained by 5 h of hydrothermal processing are too long and dense to disperse.

### The photocatalytic degradation mechanism of ZnO nanorod

The possible photocatalytic degradation mechanism of as-prepared ZnO nanorod is proposed and shown in Figure S17. Under the irradiation of visible light, ZnO nanorods are excited to generate photogenerated electrons and holes, where photogenerated electrons will migrate from the valence band (VB) to the conduction band (CB) of ZnO nanorods, leaving holes at the valence band. The electrons enriched in the conduction band of ZnO nanorods will react with O^2^ in the solution to generate ⋅O^2^ active radical to further degrade the pollutant rhodamine B. At the same time, the holes in the valence band of ZnO nanorods can react with OH^⋅−^/H_2_O to generate active ⋅OH radicals. The ⋅OH radicals and holes act together to degrade the pollutant rhodamine B. After the reactions between the active substances and pollutants, rhodamine B is finally decomposed into less if no harmful small molecules, completing the photodegradation^24–28^. The possible photocatalytic reaction in this study is shown in the following formula:

$${\text{ZnO}} + {\text{ hv }} \to {\text{ZnO}} + {\text{ e}}^{ - } + {\text{ h}}^{ + }$$

$${\text{e}}^{ - } + {\text{ O}}_{2} \to \, \cdot {\text{O}}_{2}{^{ - }}$$

$${\text{h}}^{ + } + {\text{ OH}}^{ - } /{\text{H}}_{{2}} {\text{O }} \to \cdot {\text{OH}}$$

$${\text{RhB }} + {\text{ h}}^{ + } /\cdot {\text{OH}}/\cdot {\text{O}}_{{2}}{^{ - }} \to {\text{ CO}}_{{2}} + {\text{H}}_{{2}} {\text{O}}$$

## Conclusion

In conclusion, we have shown that zinc acetate doped on electrospun nanofiber is a cost-efficient yet effective way of preparing ZnO nanocrystals and nanorods. The PVDF nanofiber mat acts as an excellent template for the in-situ growth of nano ZnO to give well-defined and uniformly-dispersed morphology. This brings advantages on the photocatalytic properties of the nano ZnO, as shown by the much improved photocatalytic degradation of Rhodamine B. The material design and synthesis in this work provides an alternative and useful approach for the efficient preparation of functional nanocrystals with controllable morphology and tunable properties that can be readily scaled up for vast production.

## Supplementary Information

Supplementary Information.
