# Supplementary material for: Nanofiber template-induced preparation of ZnO nanocrystal and its application in photocatalysis
Source: Sci Rep. 2021 Oct 27;11:21196. doi: 10.1038/s41598-021-00303-9 (PMC8551286; doi:10.1038/s41598-021-00303-9)
Supplement: Supplementary file 1 — Supplementary Information. [file 41598_2021_303_MOESM1_ESM.docx]

**Supporting information for**

**Nanofiber Template-induced Preparation of ZnO Nanocrystal and its Application in Photocatalysis**

*Mingyi Chen* ^a#^*, Peng Liu* ^b#^*, Ji-Huan He* ^b^*, Hsing-Lin Wang* ^a^*, Haonan Zhang^a^, Xin Wang** ^c^*, Rouxi Chen** ^a^

^a^ Department of Materials Science and Engineering, Southern University of Science and Technology, Shenzhen, 518055 China

^b^ National Engineering Laboratory for Modern Silk, College of Textile and Clothing Engineering, Soochow University, Suzhou, 215123 China

^c^ Songshan Lake Materials Laboratory, Dongguan, 523808 China

**Table S1. Summary of polymers, solvents and reagents used in the study**

| Name | [Grade](javascript:;) | Molecular weight (g/mol) | Supplier |
| --- | --- | --- | --- |
| Polyvinylidene fluoride (PVDF) | AR | 400,000 | J&K Chemical Ltd. |
| N,N-Dimethylformamide (DMF) | AR | 73.09 | Chinasun Specialty Products Co., Ltd. |
| Acetone | AR | 58.08 | Chinasun Specialty Products Co., Ltd. |
| Zinc Acetate (C_4_H_6_O_4_Zn) | AR | 183.48 | Shanghai Aladdin Biochemical Technology Co., Ltd. |
| Hexamethylene Tetramine (C_6_H_12_N_4_) | AR | 140.19 | Sinopharm Chemical Reagent Co., Ltd. |
| Zinc Chloride (ZnCl_2_) | CP | 136.30 | Sinopharm Chemical Reagent Co., Ltd. |
| Rhodamine B | HPLC | 479.01 | Shanghai Aladdin Biochemical Technology Co., Ltd. |
| Ammonia (NH_3_·H_2_O) | GR | 17.03 | Sinopharm Chemical Reagent Co., Ltd. |

**Table S2. Recipe of spinning solution**

| Name | PVDF (g) | Zn(CH_3_COO)_2_ (g) | DMF (g) | CH_3_COCH_3_ (g) |
| --- | --- | --- | --- | --- |
| N10 | 1.50（5%） | 0.45 | 19.95 | 8.55 |
| N20 | 3.00（10%） | 0.90 | 18.90 | 8.10 |
| N30 | 5.00（15%） | 0 | 19.80 | 8.50 |
| N31 | 5.00（15%） | 0.50 | 19.80 | 8.50 |
| N32 | 5.00（15%） | 1.50 | 19.80 | 8.50 |
| N33 | 5.00（15%） | 2.50 | 19.80 | 8.50 |
| N34 | 5.00（15%） | 3.50 | 19.80 | 8.50 |
| N40 | 7.00（19%） | 2.10 | 20.90 | 8.90 |

**Preparation of PVDF/Zn(CH_3_COO)_2_ nanofiber membrane**

The PVDF/Zn(Ac)_2_ nanofiber membranes with different concentrations (5 wt.%, 10 wt.%, 15 wt.%, 19 wt.%) were characterized by SEM, as shown in Figure S1. The uniformity of nanofiber membrane is optimal when the spinning solution concentration is 15 wt.%. In addition, the optimal PVDF/Zn(Ac)_2_ nanofiber membranes with different concentrations of zinc acetate are characterized by SEM, as shown in Figure S2. The results show that the uniformity and mechanical properties of membrane are excellent when the mass ratio of PVDF to zinc acetate in the solution is 10:3.


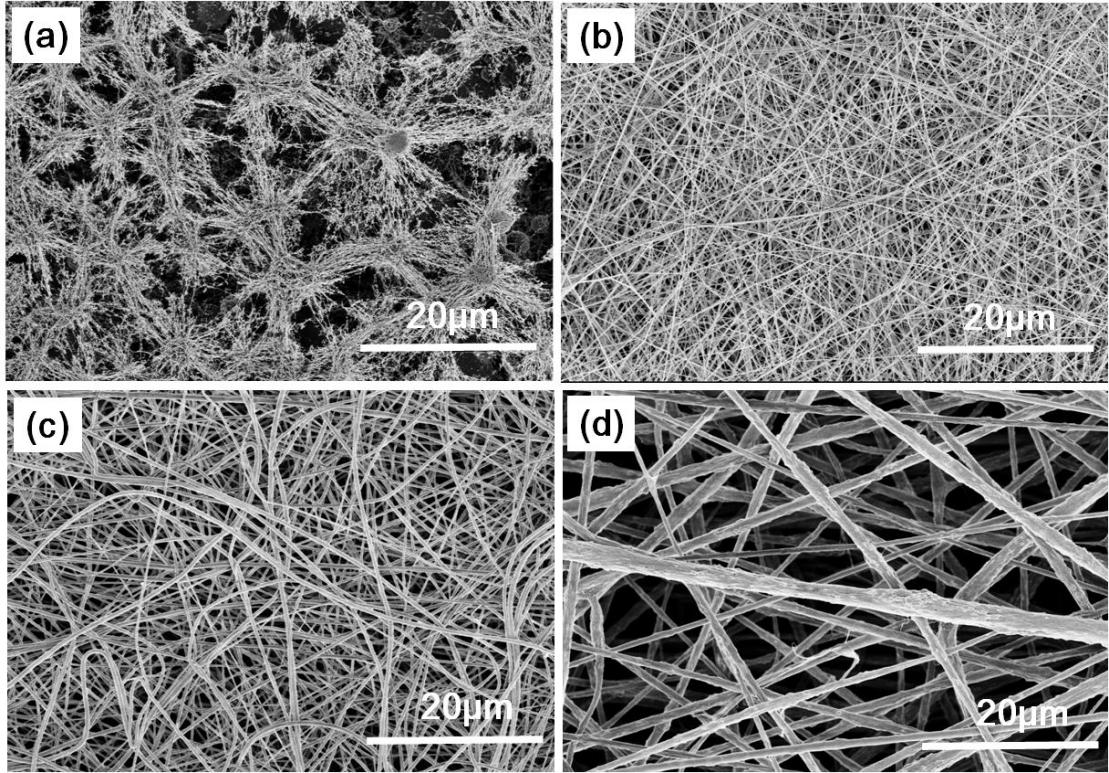


**Figure S1.** SEM images of PVDF/Zn(Ac)_2_ nanofiber mat electrospun from solution of different concentrations: (a) 5 wt.%, (b)10 wt.%, (c)15 wt.% and (d) 19 wt.%.


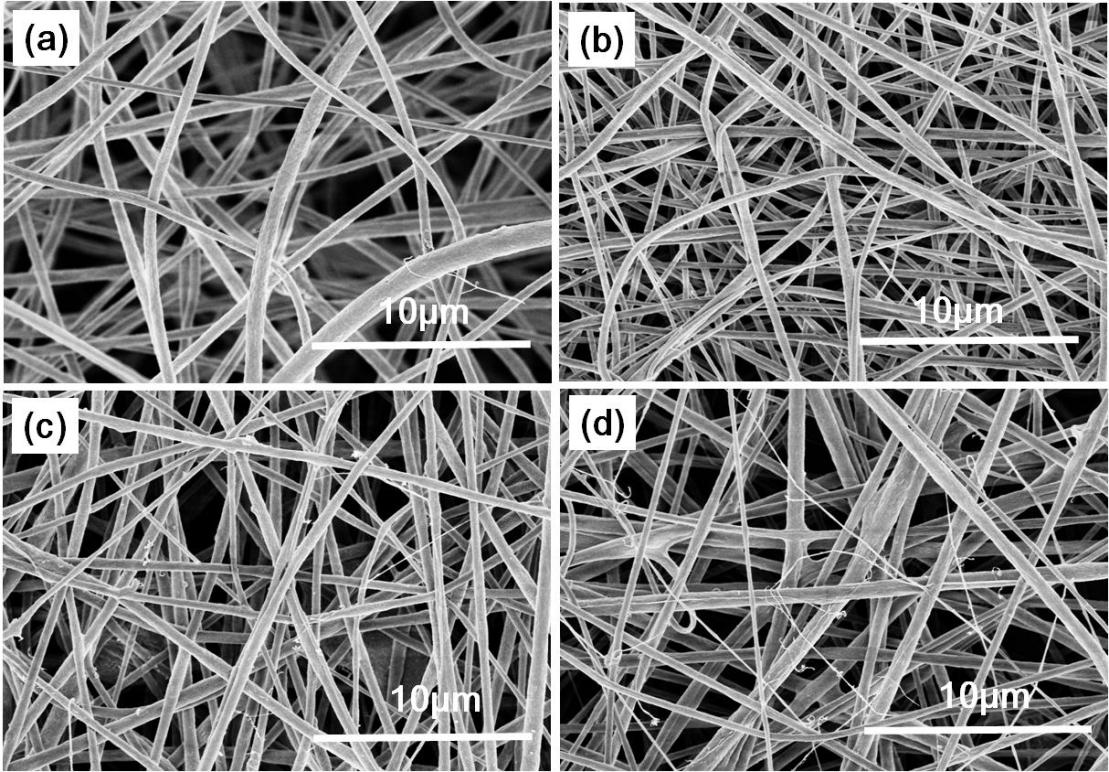


**Figure S2.** SEM images of PVDF/Zn(Ac)_2_ nanofiber mat electrospun from solutions with different zinc acetate content, m(PVDF)/m(Zn(Ac)_2_): (a) 10:1, (b) 10:3, (c) 10:5, (d) 10:7.

**ZnO-loaded PVDF nanofiber membrane**

The PVDF/Zn(Ac)_2_ nanofiber membrane was thermally treated from 60 °C to 200 °C at an interval of 20 °C for 24 h. The as-prepared nanofiber was examined by SEM (Figure S3) and TEM (Figure S4) observation, followed by mechanical and photocatalytic property measurements.

The PVDF/Zn(Ac)_2_ fiber membranes thermally treated at 140 °C for 1h, 12h, and 36h were observed by SEM (Figure S5) and subject to mechanical test. The results indicate that the heat treatment temperature has a more critical effect on the formation of ZnO crystals and the morphology of PVDF/Zn(Ac)_2_ nanofiber membranes, although the heat treatment time will affect the size and quantity of ZnO crystals. Thermal treatment at a temperature over 140 °C may cause the zinc acetate to be hydrolyzed or decomposed into low-crystalline nano-ZnO, resulting in increase in tensile strength and decrease in the elongation at break.


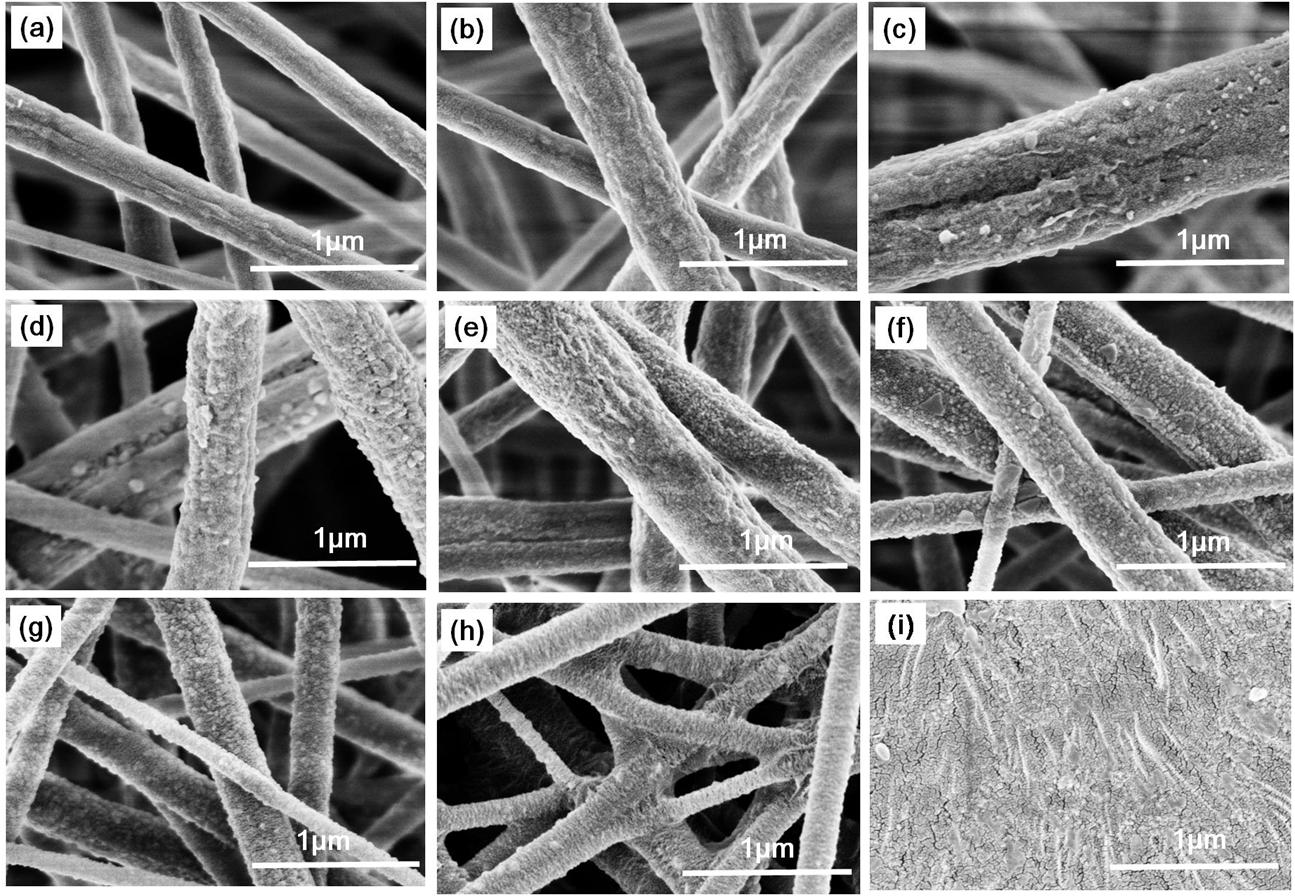


**(e)**

**Figure S3.** SEM images of PVDF/Zn(Ac)_2_ nanofiber mat with different thermal treatments for 24 h. (a), no thermal treatment, (b)-(i), thermally treated at 60 °C, 80 °C, 100 °C, 120 °C, 140 °C, 160 °C, 180 °C and 200 °C.

**Figure S4.** TEM images of PVDF/Zn(Ac)_2_ nanofiber mat with different thermal treatments for 24 h. (a), TEM image without thermal treatment. (b)-(i), TEM images thermally treated at 60 °C, 80 °C, 100 °C, 120 °C, 140 °C, 160 °C, 180 °C and 200 °C.

**Figure S5.** SEM images of PVDF/Zn(Ac)_2_ nanofiber mat thermally treated at 140 °C for 1 h (a), 12 h (b) and 36 h (c).

**Factors affecting the growth of ZnO nanorods**

The solution prepared by ZnCl_2_, HMTA and ammonia water is used as the growth solution for ZnO nanostructures, and the effects of the concentration of crystal growing solution, volume of ammonia solution, hydrothermal reaction temperature and time, and the heat treatment film at different temperatures on the highly oriented ZnO nanorods grown on the fiber were discussed.

The influence of the M(ZnCl_2_:HMTA) molar ratio on the structure of ZnO nanorods was studied, as shown in Figure S6. Four M(ZnCl_2_:HMTA) ratios were employed: 1:3, 1:2, 1:1 and 2:1. The solution with 5 mL of ammonia and a total volume of 100 mL was subject to hydrothermal reaction for 3 h.


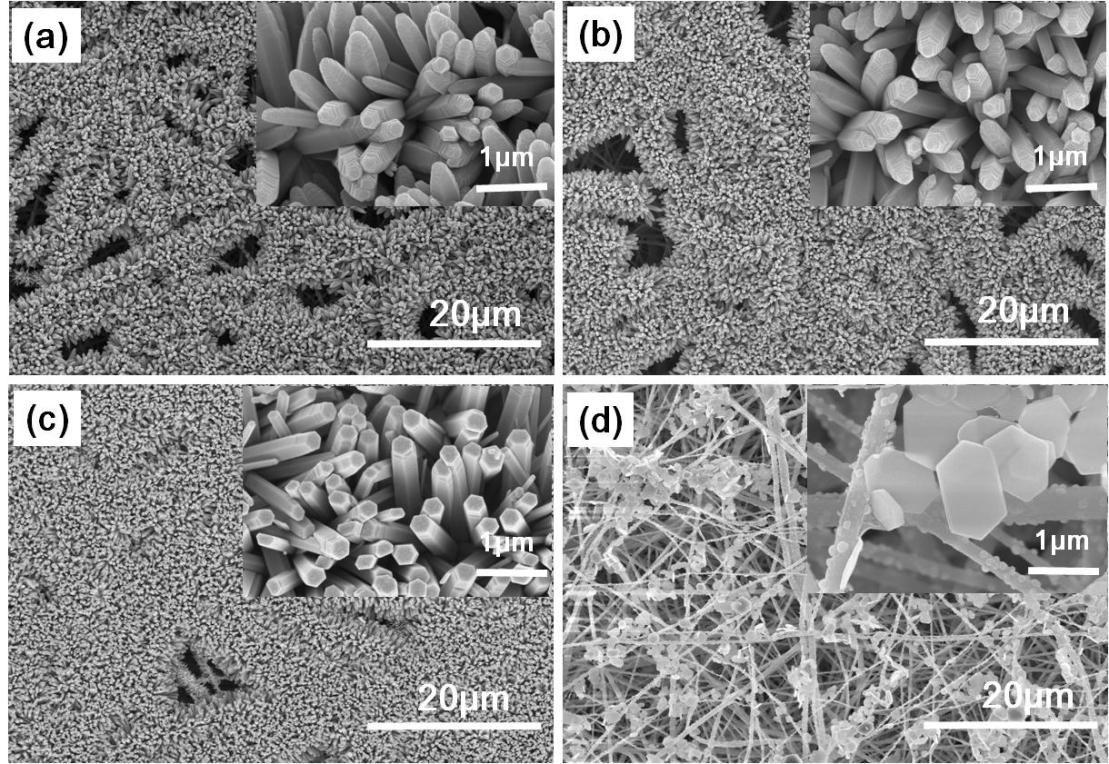


**Figure S6**. SEM images of ZnO nanorods from growing solution with M(ZnCl_2_):M(HMTA) molar ratio of 1:3 (a), 1:2 (b), 1:1 (c) and 2:1 (d).

The influence of hydrothermal temperature on the nanostructure of ZnO was investigated with a ZnCl_2_:HMTA molar ratio of 1:1 at a total concentration of 0.1 M for 3 h, as shown in Figure S7.


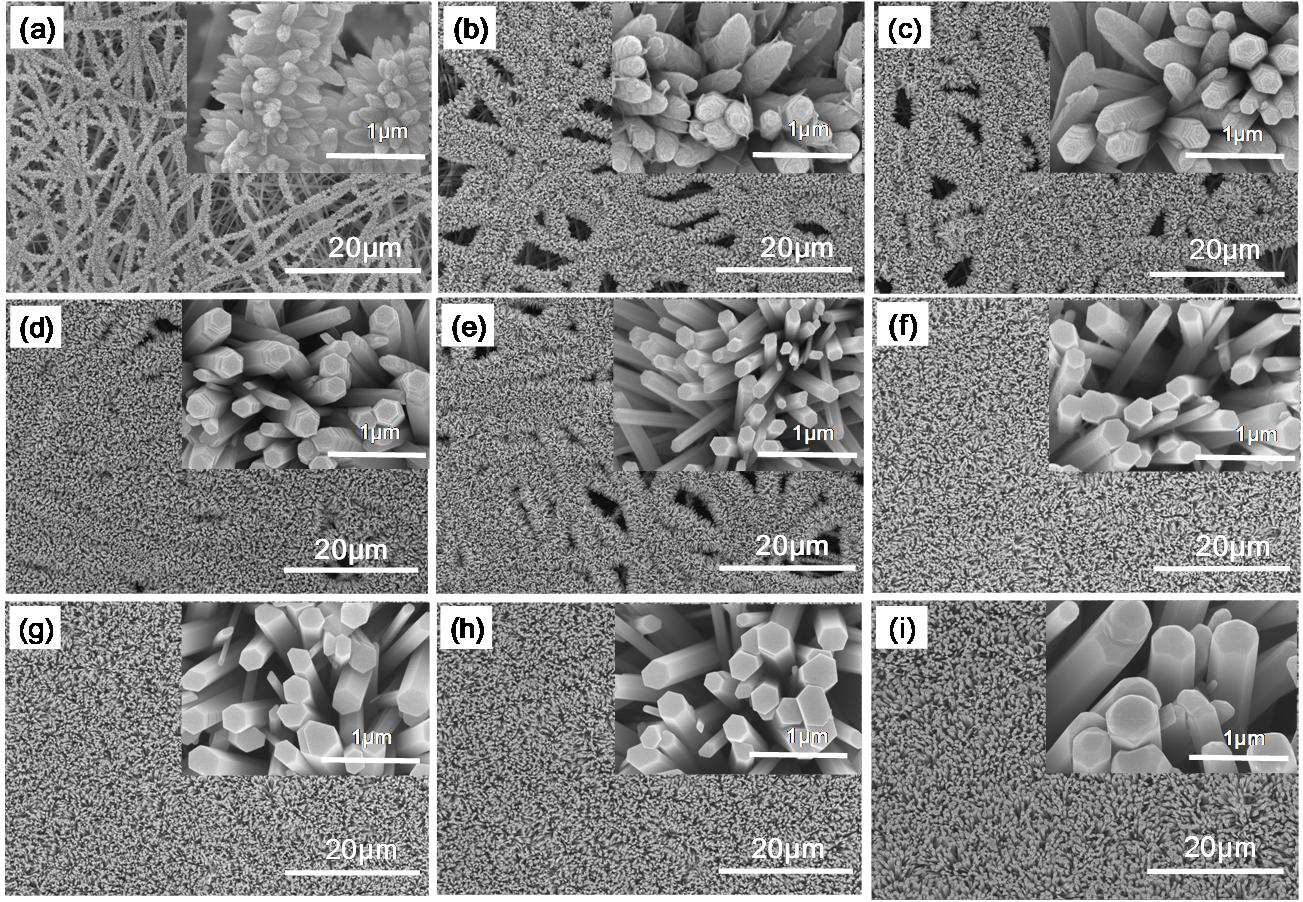


**Figure S7**. SEM images of ZnO nanorods prepared from hydrothermal temperature of 50 °C (a), 60 °C (b), 70 °C (c), 80 °C (d), 100 °C (e), 110 °C (f), 120 °C (g), 130 °C (h) and 140 °C (i).

Influence of volume of ammonia solution on the nanostructure of ZnO was studied, as shown in Figure S8.

**Figure S8**. SEM images of ZnO nanorods prepared from growing solutions with ammonia volume of 0 mL (a), 1 mL (b), 2 mL (c), 3 mL (d), 4 mL (e) and 7 mL (f).

The nanostructure of ZnO prepared from different growing time was compared, as shown in Figure S9.


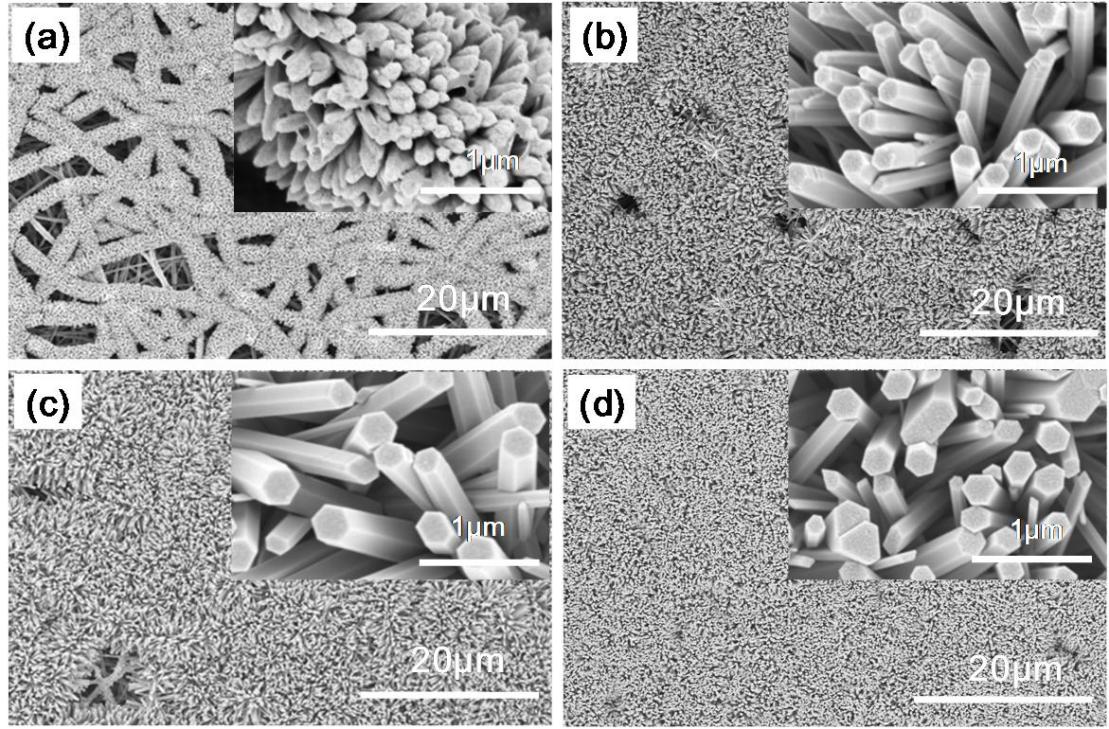

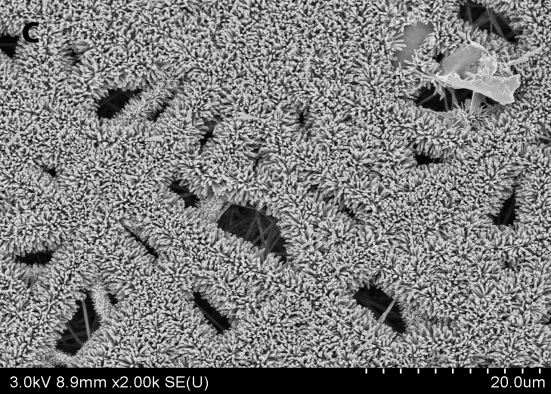


**Figure S9**. SEM images of ZnO nanorods hydrothermally prepared for 1 h (a), 3 h (b), 5 h (c) and 12 h (d).

The morphologies of ZnO prepared on nanofiber mat pre-treated for 18 h at different temperature are shown in Figure S10.

**Figure S10**. SEM images of ZnO nanorods prepared from PVDF/Zn(Ac)_2_ nanofiber mats without previous thermal treatment (a), and with thermal treatment at 60 °C (b), 80 °C (c), 140 °C (d) and 200 °C (e). SEM image of ZnO nanofilm prepared on aluminum foil (f).

**Growing behavior of ZnO nanorods**

The untreated PVDF/Zn(Ac)_2_ nanofiber mat and the PVDF/Zn(Ac)_2_ nanofiber mats with ZnO nanorods were subjected to TGA and DSC measurements, as shown in Figures S11 and S12. The weight loss, exothermic and endothermic peaks of the four nanofiber mats demonstrate that the oxidative decomposition of zinc acetate first leads to ZnO crystals, generating an obvious endothermic peak in DSC, followed by complete oxidative decomposition and generation of new substance along with liberation of heat. The weight loss in this stage is mainly attributed to the complete oxidative decomposition of the fiber and the oxidative decomposition of all zinc acetate into highly crystalline ZnO. Moreover, the thermally treated nanofiber mats are found to give more ZnO nanorods, whereas the weights of the ZnO nanorods obtained from zinc acetate coated nanofiber mat and the blended nanofiber mat are similar.

**Figure S11**. TGA thermograms of nanofibers treated under different conditions. Black, pristine PVDF/Zn(Ac)_2_ nanofiber mat. Blue, ZnO nanorods prepared on pristine PVDF/Zn(Ac)_2_ nanofiber mat. Red, ZnO nanorods prepared on thermally treated PVDF/Zn(Ac)_2_ nanofiber mat. Green, ZnO nanorods prepared on thermally treated PVDF nanofiber mat coated with Zn(Ac)_2_.

**Figure S12.** DSC curves of PVDF composite nanofiber mats. Black, pristine PVDF/Zn(Ac)_2_ nanofiber mat. Blue, ZnO nanorods prepared on pristine PVDF/Zn(Ac)_2_ nanofiber mat. Red, ZnO nanorods prepared on thermally treated PVDF/Zn(Ac)_2_ nanofiber mat. Green, ZnO nanorods prepared on thermally treated PVDF nanofiber mat coated with Zn(Ac)_2_.

The XRD diffractograms of the ZnO and PVDF composite nanofiber mats is shown in Figure S13. Among all the products, only the hydrothermally treated PVDF/Zn(Ac)_2_ fiber mat shows nine obvious diffraction peaks that correspond to the crystalline structure of ZnO. In addition, the peak intensity of (002) is somewhat greater than that of (101), indicating that the growth of the hydrothermally synthesized ZnO nanorods with a hexagonal wurtzite structure is primarily along the c-axis.

**Figure S13**. XRD diffractograms of ZnO and PVDF composite nanofiber mats. Black, pristine ZnO prepared from hydrothermal reaction. Blue, ZnO nanorods prepared from hydrothermal reaction of PVDF/Zn(Ac)_2_. Pink, PVDF/Zn(Ac)_2_ nanofiber mat thermally treated at 140 °C. Green, pristine PVDF/Zn(Ac)_2_ nanofiber mat. Red, neat PVDF nanofiber mat.

**Photocatalytic performance**

To examine the effect of ZnO nanorods on photocatalytic degradation of Rhodamine B, two groups of experiment were performed, as shown in Figure S14. The first group shows the comparison between the ZnO nanorod-loaded PVDF nanofiber membranes with different weights (1 g, 2 g and 3 g) and pristine ZnO powder (1 g) synthesized by hydrothermal method, whereas the second group shows the difference between the ZnO nanorod-loaded PVDF nanofiber membranes with the same weight (2 g) but different hydrothermal time (1 h, 3 h and 5 h). Subsequently, the nanofiber membranes with the powder of these two experiments are added to the rhodamine B solution with a concentration of 10 mg/L and a total volume of 30 mL.

**Figure S14.** Photocatalytic degradation of rhodamine B by PVDF/Zn(Ac)_2_ nanofiber mat and ZnO. Red, untreated PVDF/Zn(Ac)_2_ nanofiber mat. Blue, PVDF/Zn(Ac)_2_ nanofiber mat thermally treated at 140 °C. Pink, pristine ZnO prepared from burning at 600°C.

**Figure S15.** Photocatalytic degradation of Rhodamine B by ZnO nanorods. Degradation catalyzed by ZnO nanorod-loaded PVDF nanofiber membranes with different weights and by pristine ZnO powder.

**Figure S16.** Photocatalytic degradation of Rhodamine B by ZnO nanorods. Degradation catalyzed by 2 g of ZnO nanorod-loaded PVDF nanofiber membranes with different hydrothermal reaction time.

**Photocatalytic degradation mechanism of ZnO nanorod**

Under the irradiation of visible light, ZnO nanorods are excited to generate photogenerated electrons and holes, and their photogenerated electrons will migrate from the valence band (VB) to the conduction band (CB) of ZnO nanorods, leaving holes at the valence band. The electrons enriched in the conduction band of ZnO nanorods will react with O_2_ in the solution to generate • O^2^ active radical to further degrade the pollutant RHB. At the same time, the holes in the valence band of ZnO nanorods can react with Oh − / H_2_O to generate active radical • OH, • OH and holes act together to degrade the pollutant RhB. In conclusion, after a series of reactions between its active substances and pollutants, RhB is finally decomposed into harmless small molecular substances to realize degradation. The possible photocatalytic reaction pathway in this work is shown in the following figure.

**Figure S17.** Proposed photocatalytic degradation mechanism of ZnO nanorod
